# Comparative Evaluation of the Effect of Metformin and Insulin on Gut Microbiota and Metabolome Profiles of Type 2 Diabetic Rats Induced by the Combination of Streptozotocin and High-Fat Diet

**DOI:** 10.3389/fphar.2021.794103

**Published:** 2022-01-03

**Authors:** Nan Hu, Qi Zhang, Hui Wang, Xuping Yang, Yan Jiang, Rong Chen, Liying Wang

**Affiliations:** ^1^ Department of Pharmacy, The Third Affiliated Hospital of Soochow University/The First People’s Hospital of Changzhou, Changzhou, China; ^2^ Department of Pharmacy, Changzhou No. 7 People’s Hospital, Changzhou, China; ^3^ Department of Pathology, The Third Affiliated Hospital of Soochow University/The First People’s Hospital of Changzhou, Changzhou, China

**Keywords:** type 2 diabetes, metformin, insulin, microbiota, metabolome, bile acids

## Abstract

Lately, an increasing number of studies have investigated the relationship between metformin and gut microbiota, suggesting that metformin exerts part of its hypoglycemic effect through the microbes. However, its underlying mechanism remains largely undetermined. In the present study, we investigated the effects of metformin on gut microbiota and metabolome profiles in serum and compared it with insulin treatment in rats with type 2 diabetes mellitus (T2DM). Diabetic rats (DM group) were induced by a combination of streptozotocin and high-fat diet (HFD). After 7 days, DM rats were treated with metformin (MET group) or insulin (INS group) for 3 weeks. The 16S rRNA sequencing of the gut microbiota and non-targeted metabolomics analysis of serum were conducted. A total of 13 bile acids (BAs) in serum were further determined and compared among different groups. The rat model of T2DM was well established with the typical diabetic symptoms, showing significantly increased blood glucose, AUC of OGTT, HOMA-IR, TC, TG, LDL-C and TBA. Metformin or insulin treatment could ameliorate symptoms of diabetes and partly recover the abnormal biochemical indicators. Compared with DM rats, the relative abundances of 13 genera were significantly changed after metformin treatment, while only three genera were changed after insulin treatment. The metformin and insulin treatments also exhibited different serum metabolome profiles in T2DM rats. Moreover, 64 differential metabolites were identified between MET and DM groups, whereas 206 were identified between INS and DM groups. Insulin treatment showed greater influence on amino acids, glycerophospholipids/glycerolipids, and acylcarnitine compared with the metformin treatment, while metformin had an important impact on BAs. Furthermore, metformin could significantly decrease the serum levels of CA, GCA, UDCA, and GUDCA, but increase the level of TLCA in DM rats. Insulin treatment significantly decreased the levels of CA, UDCA, and CDCA. Besides, several metabolites in serum or microbiota were positively or negatively correlated with some bacteria. Collectively, our findings indicated that metformin had a stronger effect on gut microbiota than insulin, while insulin treatment showed greater influence on serum metabolites, which provided novel insights into the therapeutic effects of metformin on diabetes.

## Introduction

As a chronic metabolic disease with complex pathogenesis, type 2 diabetes mellitus (T2DM) refers to a spectrum of systemic illnesses related to glucose metabolism, lipid metabolism, and amino acid metabolism. Moreover, T2DM often has high rates of death and disability, and it is accompanied by severe complications. For more than 2 decades, metformin is a first-line treatment regimen to increase insulin sensitivity in T2DM patients although its underlying mechanisms of action remain largely undetermined. It is believed that metformin improve patients’ hyperglycemia by suppressing hepatic gluconeogenesis, decreasing hepatic glucose output, elevating glucose uptake and utilization in peripheral tissues, and enhancing the energy metabolism in several organs, such as muscle, fat, and liver through activating of AMP-activated protein kinase (Kristófi and Eriksson, 2021). The concentration of metformin in the bowel is 100–300 times greater compared with the serum, and about 50% of its intake is detected in the stool. The half-life of metformin is approximately 3–4 h once orally administered, which is significantly shorter than the duration of its hypoglycemic effect. Besides, metformin can not decrease blood glucose when intravenously administered. The above-mentioned findings all indicate that metformin has key impacts on the digestive tract.

Recently, with the advance of detection technology, it has been found that gut microbiota plays a fundamental role in the pathogenesis of diabetes. Accordingly, a great deal of attention has been paid to the relationship between metformin and gut microbiota. We have previously reviewed the literature concerning the effects of metformin on the gut microbiota of various species, including mice, rats, and humans with obesity or T2DM, and the compositional changes of the gut microbiota have been summarized. Accumulating evidence has indicated that metformin may change the composition of gut microbiota, through which its hypoglycemic effects are exerted (Zhang and Hu, 2020). Nevertheless, it remains largely unknown how metformin alters the gut microbiota.

To clarify the complex interaction between microbial ecosystems and host, it is necessary to adopt comprehensive analytical methods that capture the dynamic interplays among metformin, gut microbiota and diabetes. Metabolomics can determine alterations in absolute and/or relative contents of hundreds to thousands of small elements in blood and tissue, and offer valuable insights into disease diagnosis and the mechanisms of pathogenesis and drug intervention. Several metagenomic and metabolomic methods have been exploited to evaluate the phenotype of diabetic individuals and to represent decisive metabolic processes. Nevertheless, the association between gut microbiota and metformin-regulated metabolites remains largely unclear in the pathogenesis of diabetes.

In our current work, 16S rRNA gene sequencing analysis was used to assess the alterations of the gut microbiota in T2DM rats induced by a combination of streptozotocin (STZ) and high-fat diet (HFD). Moreover, we also evaluated the intervention effect of metformin and insulin. Besides, differential metabolites in serum were identified by non-targeted and targeted metabolomics analyses. Furthermore, the interplay between the gut microbiota and host metabolism was investigated to unravel the mechanism of metformin in the treatment of T2DM.

## Materials and Methods

### Materials and Reagents

Methanol, acetonitrile and formic acid of HPLC-grade were provided by Merck (Dannstadt, Germany). STZ, reference bile acid (BA) standards, including cholic acid (CA), glycocholic acid (GCA), deoxycholic acid (DCA), chenodeoxycholic acid (CDCA), ursodeoxycholic acid (UDCA), glycochenodeoxycholic acid (GCDCA), taurocholic acid (TCA), tauroursodeoxycholic acid (TUDCA), glycoursodeoxycholic acid (GUDCA), taurochenodeoxycholic acid (TCDCA), taurodeoxycholic acid (TDCA), lithocholic acid (LCA), and taurolithocholic acid (TLCA), and isotope internal standards were supplied by Sigma-Aldrich (St. Louis, MO, United States). Metformin (purity > 95%) was purchased from Aladdin Reagent Co., Ltd. (China). Insulin (NovoLet®N) was applied by Novo Nordisk Pharmaceutical Industries, Inc. Normal and high-fat chow were obtained from TROPHIC Animal Feed High-Tech Co., Ltd. (Nantong, China). Deionized water was purified using a MilliQ system (Millipore Corporation, MA, United States).

### Animals

Sprague-Dawley rats (male, 110–150 g) were purchased from Cavens Experimental Animal Co., Ltd. (Changzhou, China), and the animals were bred in a facility under the controlled conditions (22–24°C, relative humidity 55–60%, and a 12-h light/dark photoperiod). The rats were given free access to water and food and acclimatized to the animal facility for 3 days before the experiment.

### Animal Experiments

T2DM was induced by a combination of low-dose STZ via intraperitoneal injection and HFD as previously described (Wang et al., 2019). Briefly, the rats were divided into CON (*n* = 6) and DM (*n* = 40) groups. The rats in the CON group were fed on normal chow, while DM rats were fed on HFD containing 15% lard (w/w), 20% sucrose, 5% sesame oil, 2.5% cholesterol, and 57.5% normal chow for 5 weeks. Following overnight fasting, DM rats were intraperitoneally injected with a single dose of STZ (35 mg/kg). CON rats only received the vehicle solution. At 7 days after the administration of STZ, the level of fasting blood glucose (FBG) was measured. Only rats with an FBG level higher than 11.1 mM were considered as successful DM rats and used for the subsequent experiments. The DM rats were then randomly divided into three groups: 1) DM group (*n* = 7), continually fed with HFD; 2) MET group (*n* = 7), fed with HFD and intragastrically administered with 300 mg/kg body weight metformin once daily for 3 weeks; and 3) INS group (*n* = 7), fed with HFD and subcutaneously injected with insulin (2–4 U/day) according to glucose levels for 3 weeks. FBG and body weight were monitored and recorded during the experiments. Animal protocols complied with institutional guidelines for the care and the use of laboratory animals and were authenticated by the Ethics Committee of the Third Affiliated Hospital of Soochow University.

### Oral Glucose Tolerance Test and Sample Collection

OGTT was conducted 3 days before the end of the animal experiment. Briefly, 12-h fasting-adapted rats were orally administered with glucose solution (2 g/kg). The blood glucose levels were measured at 0, 15, 30, 60, and 120 min after the glucose administration, the corresponding curves were plotted, and the areas under the curve (AUCs) of OGTT were calculated. After 9 weeks, rats were sacrificed under ether anesthesia, and blood specimens were harvested from the abdominal aorta. The blood samples were allowed to stand at room temperature for 2 h and centrifuged at 3, 500 rpm for 10 min. The liver and colon were collected. The contents of the colon were placed in sterile Falcon tubes, followed by storage at −80°C before DNA isolation. An automatic biochemistry analyzer (AU5800, Beckman Coulter, United States) was adopted to analyze the serum biochemical parameters, including fasting serum glucose (GLU), total cholesterol (TC), triglycerides (TGs), high-density lipoprotein cholesterol (HDL-C), low-density lipoprotein cholesterol (LDL-C), total bile acid (TBA), urea, creatinine (Cr), alanine aminotransferase (ALT), and aspartate aminotransferase (AST). Serum insulin was measured using an electrochemiluminescence immunoassay. The homeostasis model of assessment for insulin resistance index (HOMA-IR) was calculated as [fasting serum glucose (mmol/L) × fasting serum insulin (mIU/L)]/22.5.

### Histological Assessment

The liver and colon were collected, followed by fixation in 10% buffered formaldehyde. After being rinsed with tap water, the specimens were dehydrated in increasing concentrations of alcohol (70% alcohol for 1 h, then 96% alcohol for 1 h three times). The paraffin-embedded tissues were cut into 4-mm sections using a microtome (Leica RM 2015, Germany), followed by hematoxylin-eosin (H&E) staining. An Olympus CX31 microscope (Olympus Hamburg, Germany) was adopted to examine the sections.

### Gut Microbiota Analysis

An E.Z.N.A. Stool DNA Kit (Omega Bio-tek, Norcross, GA, United States) was adopted to extract microbial DNA according to the manufacturer’s instructions. The primers 338F (5′-ACT​CCT​ACG​GGA​GGC​AGC​AG-3′) and 806R (5′-GGACTACHVGGGTWTCTAAT-3′) were used to amplify the V3-V4 region of the bacterial 16S ribosomal RNA gene using a GeneAmp 9,700 thermocycler (ABI, United States) as previously described (Hu et al., 2021). The structure of the gut microbiota was assessed by dual-indexing amplification and sequencing on the Illumina MiSeq platform, followed by QIIME (version 1.6.0) bioinformatic analysis.

Raw files of Fastq format were quality-filtered by Trimmomatic and merged by FLASH based on the criteria as follows. The reads were truncated at any site receiving an average quality score <20 over a 50 bp sliding window. Sequences greater than 10 bp were amalgamated based on their overlap with no more than 2 bp. Sequences of each sample were separated according to barcodes (exactly matching) and primers (allowing 2 nucleotide mismatching), while reads consisting of ambiguous bases were discarded. A novel “greedy” algorithm that performs chimera filtering and operational taxonomic unit (OTU) clustering simultaneously was used to cluster OUT with a similarity cutoff of 97% using UPARSE (version 7.1 http://drive5.com/uparse/). The RDP Classifer algorithm (http://rdp.cme.msu.edu/) was used to analyze the taxonomy of each 16S rRNA gene sequence against the Silva (SSU123) 16S rRNA database, and the confidence threshold was set at 70%. Alpha diversity (ACE and Chao index, which were used to assess the community richness) and beta diversity were calculated using QIIME. OTUs were analyzed by unweighted UniFrac distance-metrics analysis for each sample. Principal component analysis (PCA) was then carried out according to the matrix-of-distance.

### Non-Targeted Metabolomics Analysis

Briefly, 100 μL of serum sample was mixed with 400 μL acetonitrile/methanol (v/v, 1:1) containing the internal standard of L-2-chlorophenylalanine (2 μg/ml), followed by extractions of metabolites. Subsequently, the specimens were vortexed for 30 s, sonicated for 10 min in an ice-water bath, incubated at −40°C for 1 h, and centrifuged at 10,000 rpm for 15 min at 4°C. Next, 425 μL of supernatant was dried at 37°C, and the residuals were reconstituted in 200 μL of 50% acetonitrile by sonication on ice for 10 min. The sample was then centrifuged at 12,000 rpm for 15 min at 4°C, and 75 μL of supernatant was subjected to LC/MS/MS. Equal aliquots of the supernatants from all of the samples were mixed, which were used as quality control (QC) samples.

The metabolites were separated using a UHPLC system (1,290, Agilent Technologies), which was equipped with a UPLC BEH Amide column (2.1*100 mm, 1.7 μm, Waters) coupled to a TripleTOF6600 (Q-TOF, AB Sciex) at Biotree Biotech Co., Ltd. (Shanghai, China). The mobile phase was composed of 25 mM ammonium acetate and 25 mM ammonia hydroxide in water (pH = 9.75) (A) and acetonitrile (B). The elution program was conducted as follows: 0–0.5 min, 95%B; 0.5–7.0 min, 95–65% B; 7.0–8.0 min, 65–40% B; 8.0–9.0 min, 40% B; 9.0–9.1 min, 40–95% B; 9.1–12.0 min, 95% B. The volume of injection was 1 μL (pos) or 1 μL (neg). The column temperature was maintained at 25°C. The conditions of electrospray ionization (ESI) source were set as follows: gas 1 at 60 psi, gas 2 at 30 psi, curtain gas at 35 psi, source temperature as 600°C, declustering potential at 60 V, and ion spray voltage floating (ISVF) at 5,000 V or −4,000 V in positive or negative modes, respectively.

MS raw data (wiff) files were transformed to the mzXML format by ProteoWizard and processed by R package XCMS (version 3.2). Such a process included peak deconvolution, alignment, and integration. Minfrac and cut-off values were set as 0.5 and 0.6, respectively. An in-house MS2 database was applied for the identification of metabolites. Subsequently, multivariate statistical analyses were carried out using the SIMCA-P software (version 14.1, Umetrics AB, Umea, Sweden), including PCA and OPLS-DA. The clusters, differences, and outliners in different groups were assessed using PCA, and the metabolic difference between the two groups was analyzed using the OPLS-DA. Differential metabolites were defined as those metabolites with an adjusted *p* < 0.05 and variable importance (VIP) > 1. R2 (goodness of fit parameter) and Q2 (goodness of prediction parameter) values were used for the quality evaluation of each model. Besides, cross-validation and testing with 200 permutations were adopted to avoid the over-fitting of the OPLS-DA model.

### Serum Bile Acids Measurement

To a 50 μL aliquot of each serum sample, 20 μL (100 ng/ml) of internal standard (TCA-d4, GCA-d5, CDCA-d4, DCA-d5, GCDCA-d7, and LCA-d4) and 150 μL of acetonitrile solution were added. The mixture was then vortexed for 30 s and centrifuged at 16,400 rpm for 10 min. An aliquot (100 μL) of the supernatant was diluted by 100 μL ultrapure water and then analyzed. The levels of 13 serum BAs, including TUDCA, TCA, GUDCA, GCA, TCDCA, TDCA, CA, UDCA, GCDCA, CDCA, LCA, TLCA, and DCA, were determined using a validated high-performance liquid chromatography-tandem mass spectrometry (HPLC-MS/MS) method. The chromatographic system (Jasper™ HPLC system) consisted of a vacuum degasser, a binary pump, an autosampler, and a Kinetex EVO C18 column (50 × 2.1 mm, 2.6 μm, Phenomenex, United States) and was operated at 40°C. The mobile phase consisted of water containing 0.1% formic acid and 0.5% ammonia (A) and acetonitrile (B). The gradient elution started at 25%B, increased to 35%B (0.01–4.50 min), 50%B (4.50–6.00 min), and 95%B (6.00–6.10 min), maintained at 95%B (6.10–7.40 min), and then restored to 25% (7.50–9.00 min). The flow rate was fixed at 0.4 ml/min. The injection volume was 10 μL. The AB SCIEX Triple Quad™ 4500MD mass spectrometer (Applied Biosystem Sciex, Ontario, Canada) was used for qualitative and quantitative analysis. The mass spectrometer was operated in multiple reaction monitoring (MRM) and negative ESI mode (−3500 V) with the following parameters: ion source temperature, 500°C; nebulizer gas (gas 1), nitrogen, 55 psi; turbo gas (gas 2), nitrogen, 45 psi; and curtain gas, nitrogen, 30 psi. The precursor ion and product ion mass, declustering potentials (DP), collision energies (CE), and retention time of each bile acid were summarized in Supplementary Table S1.

Among these 13 BAs, primary BAs included CA and CDCA, as well as their glycine-conjugates and taurine-conjugates, such as GCA, GCDCA, TCA, and TCDCA, while secondary BAs produced by deconjugation and/or dehydroxylation of primary BAs by gut bacteria included DCA, UDCA, and LCA, as well as their glycine-conjugates and taurine-conjugates, such as TLCA, TDCA, GUDCA, and TUDCA.

### Statistical Analysis

GraphPad Prism software v 9.0 was employed for all statistical assays. The results were expressed as mean ± SD. The difference between the two groups was compared using unpaired Student’s t-test, and multiple comparisons were performed using one-way ANOVA, followed by Dunnett’s post hoc test. The relationship between differential metabolites and the relative abundance of the intestinal microbiome at the genus level was evaluated by Pearson’s correlation analysis. Corrections of *p* values for multiple comparisons were controlled by FDR, and *p* < 0.05 was considered statistically significant.

## Results

### T2DM Modeling

**FIGURE 1:**
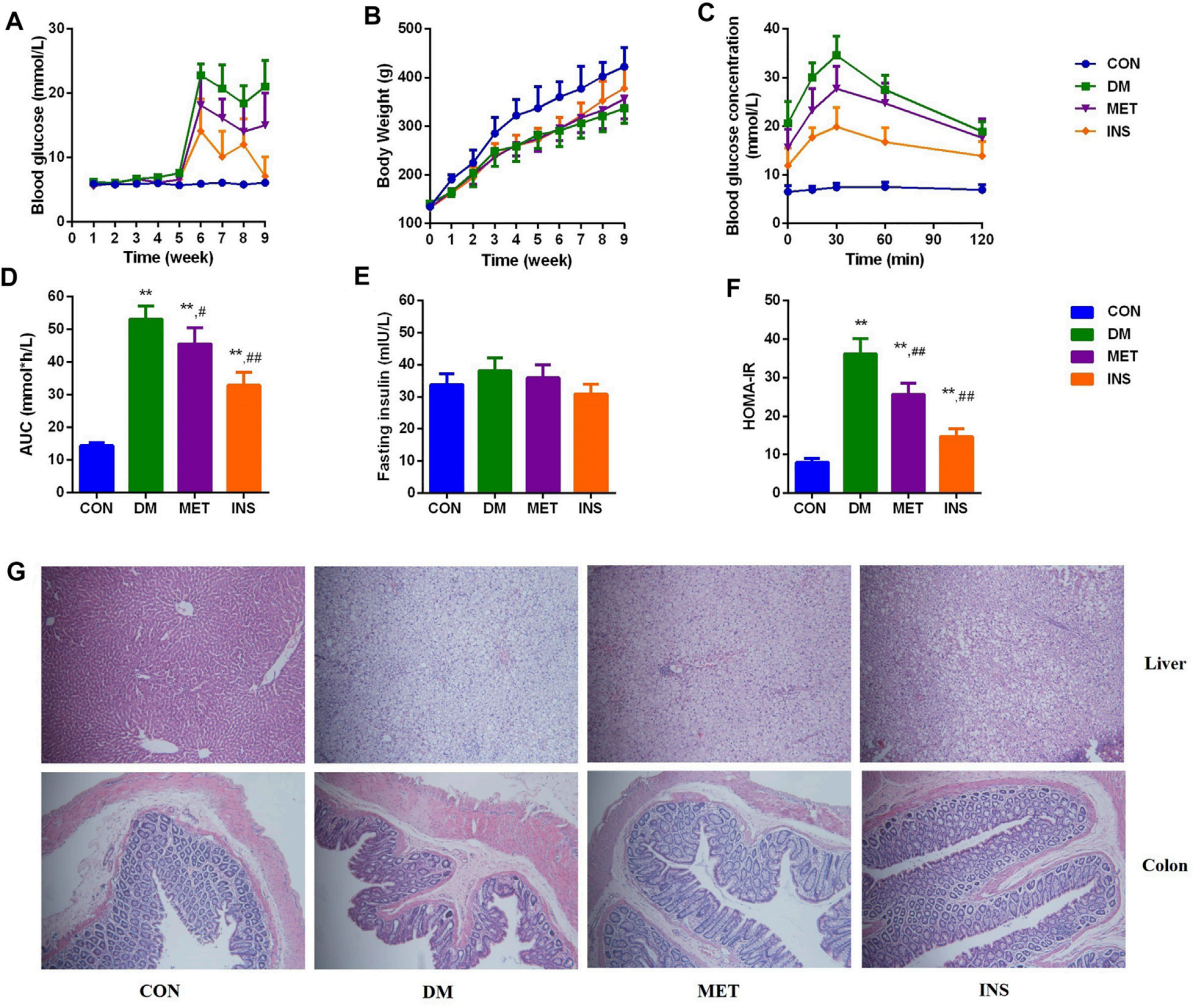
Effects of metformin and insulin on **(A)** Blood glucose; **(B)** Body weight; **(C)** Oral glucose tolerance test (OGTT); **(D)** Area under the curve (AUC) of OGTT; **(E)** Fasting insulin; **(F)** HOMA-IR index; **(G)** Histological structure of liver and colon (HE staining) *p < 0.05, **p < 0.01 vs. CON group. #*p* < 0.05, ##*p* < 0.01 vs. DM group.

After STZ injection, diabetic symptoms were observed in most rats, including polyuria, polydipsia, and polyphagia. The FBG levels of 21 rats were higher than 11.1 mM after 7 days, which were chosen for the following experiment. However, because of severe diabetes, two rats in the DM group died in the eighth and ninth weeks of the experiments. After STZ injection (the fifth week), the levels of FBG were remarkably increased in the DM group compared with the CON group. Metformin and insulin treatment could reduce the levels of FBG, and insulin exhibited higher hypoglycemic effect than metformin during the experiment (Figure 1A). Because HFD influenced the appetite of rats, the body weight of rats in the DM, MET, and INS groups was significantly decreased from the first week of the experiment. Metformin and insulin treatment could both increase the body weight of DM rats (Figure 1B). OGTT was performed in different groups, and the corresponding AUC was also analyzed (Figures 1C,D). The results of OGTT were ameliorated to some extent in the MET and INS groups. The levels of fasting insulin were comparable among CON, DM, MET and INS groups (Figure 1E). HOMA-IR index was markedly greater in the DM group compared with the CON group, implying that the islet function of the DM group was affected. The HOMA-IR index was remarkably decreased in the MET group and INS group compared with the DM group, indicating that the treatment of metformin and insulin significantly improved the insulin resistance of diabetic rats (Figure 1F).

### Histological Assessment

Histological assessments were conducted in the liver and colon of rats from different groups (Figure 1G). The liver lobule had a clear structure, and the liver cells exhibited a radial distribution around the central vein in the CON group. The structure of the liver lobule in the DM group disappeared, and the liver cord showed disordered arrangement, exhibiting widened hepatic sinusoids. Hepatocytes appeared swelling, and the size was increased. Moreover, we observed lipid droplets of different sizes in the cytoplasm with focal steatosis in hepatocytes. In the MET group, the hepatocellular cord showed a clear structure with normal liver sinusoids, mild steatosis was observed in the cytoplasm of the liver cells, and lipid droplets were decreased. The hepatocellular cord in the INS group displayed a clear structure, and mild steatosis was observed in the cytoplasm of the liver cells. The swelling of hepatocytes was relieved. Compared with the CON group, DM rats showed damaged mucosal architecture in the colon. The epithelium was slightly hyperplastic with villus atrophy. Taken together, metformin and insulin treatment significantly improved these pathological conditions.

### Biochemical Parameters

Compared with the CON group, the levels of TC, TG, and LDL-C were significantly increased in the DM group, indicating dyslipidemia in diabetic rats. Metformin and insulin could suppress the levels of TC, TG, and LDL-C in different degrees (Table 1). Compared with the CON group, the level of TBA was markedly higher in DM rats, and both metformin and insulin treatment could decrease the TBA level. There was no difference in urea, Cr, ALT, and AST among the four groups.

**TABLE 1:** Biochemical parameters.

Parameters	CON(*n* = 6)	DM(*n* = 5)	MET (*n* = 7)	INS(*n* = 7)
TC (mmol/L)	1.81 ± 0.42	7.92 ± 3.70**	6.64 ± 2.76**	6.05 ± 2.22**
TG (mmol/L)	1.26 ± 0.28	1.66 ± 0.07*	1.11 ± 0.25##	0.50 ± 0.17**,##
HDL-C (mmol/L)	0.86 ± 0.14	0.63 ± 0.27	0.62 ± 0.13**	0.44 ± 0.17**
LDL-C (mmol/L)	0.42 ± 0.14	5.36 ± 2.94**	4.26 ± 2.09**	3.45 ± 0.73**
TBA (μmol/L)	11.18 ± 7.19	20.22 ± 3.14*	15.84 ± 4.42#	10.29 ± 3.96##
Urea (mmol/L)	5.44 ± 0.65	5.21 ± 1.57	5.29 ± 1.75	4.67 ± 1.08
Cr (μmol/L)	51.00 ± 4.98	48.40 ± 10.97	53.43 ± 5.35	49.43 ± 5.38
ALT (U/L)	59.50 ± 18.43	65.80 ± 15.71	94.57 ± 60.44	62.71 ± 9.72
AST (U/L)	165.33 ± 21.42	154.40 ± 72.57	186.43 ± 54.46	135.14 ± 14.06*

Values were presented as means ± SD; TC, total cholesterol; TG, triglycerides; HDL, high-density lipoprotein; LDL, low-density lipoprotein; TBA, total bile acid; Cr, creatinine; ALT, alanine aminotransferase; AST, aspartate transaminase. *p < 0.05, **p < 0.01 vs. CON group. #*p* < 0.05, ##*p* < 0.01 vs. DM group.

### Effect of Metformin and Insulin on the Gut Microbiota

**FIGURE 2:**
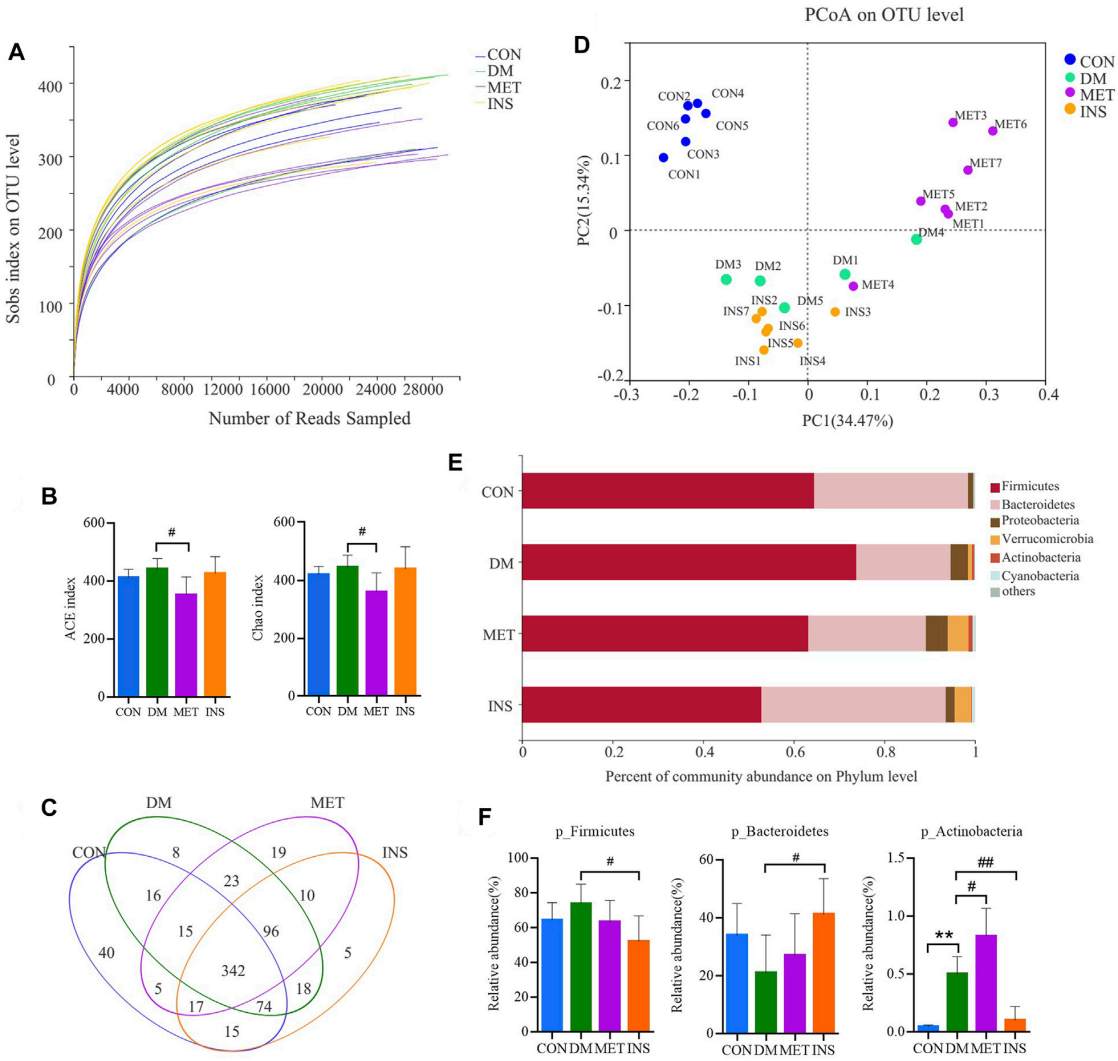
Gut microbiota response to metformin and insulin treatment. **(A)** Rarefaction curves of the gut microbiota. **(B)** ACE and Chao index. **(C)** Venn **(D)** weighted Unifrac PCoA of gut microbiota based on the OUT abundance **(E)** Relative abundance of gut microbiota in four groups at the phylum level **(F)** Relative abundances of Firmicutes, Bacteroidetes and Actinobacteria in different groups. *p < 0.05, **p < 0.01 vs CON group. #*p* < 0.05, ##*p* < 0.01 vs DM group.

A total of 1,178 012 sequences were obtained from 32 samples, and averagely 36,812 sequences were recovered for each sample and used for comparative analysis. The Good’s coverage for the observed OTUs was 99.76 ± 0.02%, and the rarefaction curves displayed clear asymptotes (Figure 2A), together indicating a near-complete sampling of the community. Figure 2B shows the ACE and Chao index of four groups, and metformin significantly decreased the ACE and Chao indexes compared with the DM group. A total of 703 OTUs were yielded from 32 samples, including 342 shared OTUs for four groups, and there were 40, 8, 19 and 5 special OTUs for the CON, DM, MET, and INS groups, respectively (Figure 2C). Weighted UniFrac PCoA distances showed separation among the CON, DM, MET, and INS groups. Based on the PCoA analysis, different trends were observed from the intestinal microbiota structure of the MET and INS groups, and both of them were clearly separated from the DM and CON groups (Figure 2D). Figure 2E shows the top six phyla in four groups. The dominant phyla included Firmicutes and Bacteroidetes. DM rats had a greater abundance of Firmicutes and a lower abundance of Bacteroidetes compared with the CON group, and thus the ratio of Bacteroidetes/Firmicutes was significantly lower in the DM (0.31) group compared with the CON (0.56) group (*p* < 0.05). Both metformin and insulin treatment could reduce the abundance of Firmicutes and elevate the abundance of Bacteroidetes in DM rats, and the ratio of Bacteroidetes/Firmicutes was 0.47 and 0.89 in the MET and INS groups, respectively. In addition, DM rats showed a significantly higher abundance of the phyla Actinobacteria (*p* < 0.01) compared with the CON group. Metformin treatment further increased such abundance, while insulin treatment decreased the abundance of Actinobacteria (Figure 2F).

**FIGURE 3:**
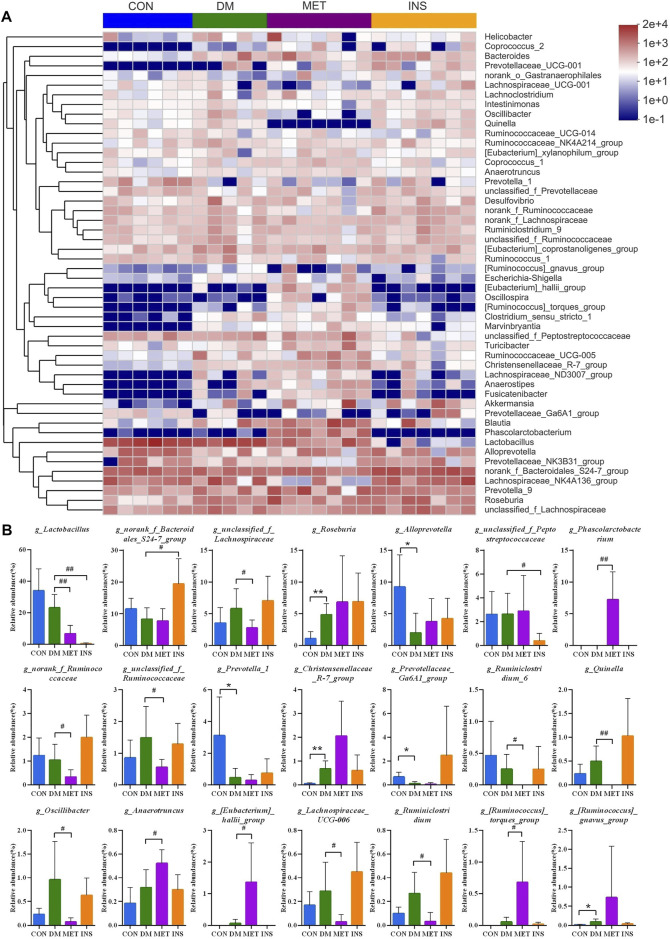
The detailed effects of metformin on the gut microbiota of diabetic rats at the genus level. **(A)** the relative abundances of 50 dominant genera in the gut microbiota of four groups are presented in a heatmap. **(B)** Relative abundances of *g_Lactobacillus*, *g_norank_f_Bacteroidales_S24-7_group*, *g_unclassified_f_Lachnospiraceae*, *g_Roseburia*, *g_Alloprevotella*, *g_unclassified_f_Peptostreptococcaceae*, *g_Phascolarctobacterium*, *g_Ruminiclostridium_9*, *g_norank_f_Ruminococcaceae*, *g_Desulfovibrio*, *g_Turicibacter*, *g_unclassified_f_Ruminococcaceae*, *g_Prevotella_1*, *g_Christensenellaceae_R-7_group*, *g_Prevotellaceae_Ga6A1_group*, *g_Ruminiclostridium_6*, *g_Quinella*, *g_Prevotellaceae_UCG-001*, *g_Oscillibacter*, *g_Clostridium_sensu_stricto_1*, *g_Anaerotruncus*, *g_(Eubacterium)_hallii_group*, *g_Lachnospiraceae_UCG-006*, *g_Ruminiclostridium*, *g_(Ruminococcus)_torques_group* and *g_(Ruminococcu)_gnavus_group* in the gut microbiota of four groups. *p < 0.05, **p < 0.01 vs. CON group. #*p* < 0.05, ##*p* < 0.01 vs. DM group.

Figure 3A shows a heatmap presenting the detailed intestinal microbiota composition (top 50) at the genus level. The relatively predominant taxa at the genus level were *Lactobacillus*, *norank_f_Bacteroidales_S24-7_group*, *Lachnospiraceae_NK4A136_group*, and *Alloprevotella*. Compared with the CON group, the abundances of *Roseburia*, *Christensenellaceae_R-7_group*, and (*Ruminococcus*)*_gnavus_group* were significantly increased, while the abundances of *Alloprevotella*, *Prevotella_1*, and *Prevotellaceae_Ga6A1_group* were significantly decreased in the DM group. After metformin treatment, the composition of intestinal microbiota changed a lot at the genus level. The abundances of *Phascolarctobacterium*, *Anaerotruncus*, (*Eubacterium*)*_hallii_group*, and (*Ruminococcus*)*_torques_group* were significantly higher, while the abundances of *Lactobacillus*, *unclassified_f_Lachnospiraceae*, *norank_f_Ruminococcaceae*, *unclassified_f_Ruminococcaceae*, *Ruminiclostridium_6*, *Quinella*, *Oscillibacter*, *Lachnospiraceae_UCG-006*, and *Ruminiclostridium* were significantly lower in the MET group compared with the DM group. However, insulin treatment showed little impact on the intestinal microbiota at the genus level. The abundance of *norank_f_Bacteroidales_S24-7_group* was increased, and the abundance of *Lactobacillu* and *unclassified_f_Peptostreptococcaceae* was decreased in the INS group compared with the DM group (Figure 3B).

### Non-targeted Metabolomics Analysis

**FIGURE 4:**
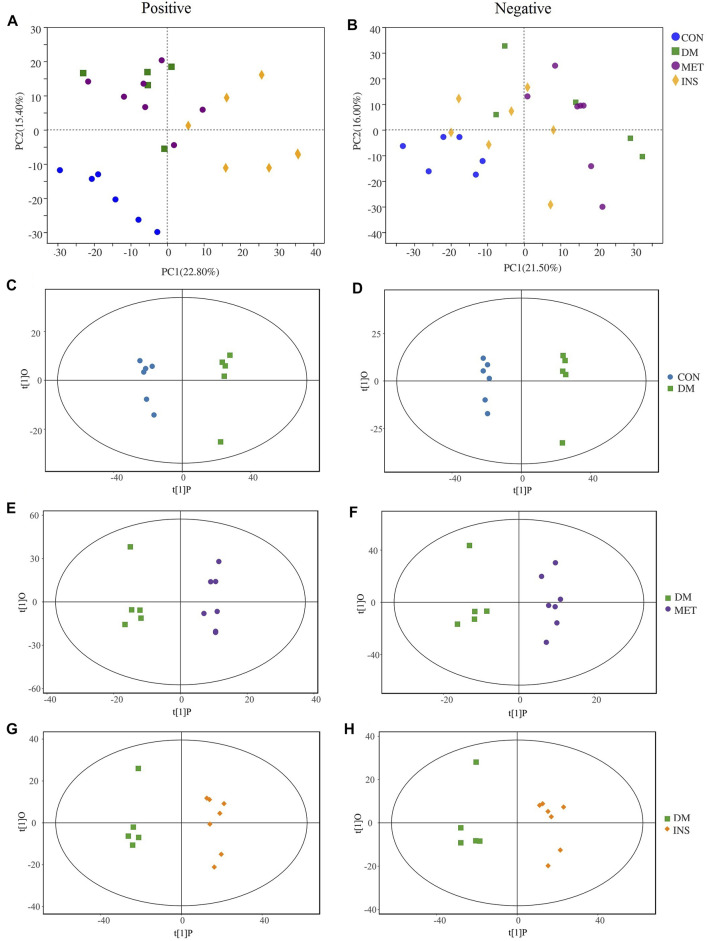
PCA and OPLS-DA score plots in positive mode and negative mode. PCA score plot of each group in positive mode **(A)** and negative mode **(B)**. OPLS-DA score plots from CON group vs DM group in positive mode **(C)** and negative mode **(D)**; MET group vs DM group in positive mode **(E)** and negative mode **(F)**; INS group vs DM group in positive mode **(G)** and negative mode **(H)**.

To further elucidate the therapeutic mechanisms of metformin and insulin on T2DM, we assessed the serum metabolites in CON, DM, MET, and INS groups according to metabolomics. We first applied the PCA model for data interpretation to explore the general trend of the four groups. The generalized separation of variations was primarily conducted according to PCA, and the variations of all groups were calculated using the OPLS-DA method based on the VIP values. Figures 4A,B show that a superior separation existed among the CON, DM, MET, and INS groups in both ESI^+^ and ESI^−^ modes. PCA score plots showed that significant differences were observed between the CON group and DM group in the positive and negative ions, indicating that the serum metabolites in T2DM rats were remarkably altered. The PCA loading diagram showed that metformin and insulin could affect the serum metabolic composition of DM rats in different degrees, indicating that the abnormal metabolism in DM rats was ameliorated after metformin and insulin treatment.

To further identify differential metabolites and to increase the number of representative latent biomarkers, we applied the OPLS-DA to distinguish the two groups. A more clear separation among different groups was achieved using the supervised OPLS-DA model. Figures 4C,D show that there was a clearly distinction between the CON group and DM group in ESI+ and ESI- modes (C: R2X = 0.802, R2Y = 0.998, Q2 = 0.863; D: R2X = 0.750, R2Y = 0.996, Q2 = 0.969). Figures 4E,F, display that the MET group and DM group were obviously separated in the ESI+ and ESI- modes (E: R2X = 0.506, R2Y = 0.996, Q2 = 0.524; F: R2X = 0.438, R2Y = 0.954, Q2 = 0.318). Figures 4G,H show that the INS group and DM group also had an obvious variation in the ESI+ and ESI- modes (G: R2X = 0.841, R2Y = 0.997, Q2 = 0.787; H: R2X = 0.843, R2Y = 0.997, Q2 = 0.751).

### Identification of Differential Metabolites

In our current work, we adopted the supervised OPLS-DA model to identify the biomarkers based on the *p* < 0.05 and VIP > 1. Next, we searched the accurate mass to charge ratio (m/z) of the positive and negative ions in the online library (http://www.hmdb.ca/) to identify the qualified elements. In addition, the potential biomarkers were surmised by the fragmentation behaviors of MS/MS. According to the criteria of *p* < 0.05 and VIP > 1, a few metabolites were identified as the latent biomarkers. Moreover, 328, 64, and 206 differential metabolites were identified among DM/CON, MET/DM, and INS/DM groups, respectively (Supplementary Table S2). Four types of (amino acids, BAs, glycerophospholipids/glycerolipids, and acylcarnitines) and 47 metabolites related to glucose metabolism were screened and identified as potential biomarkers in the MET or INS group (Table 2). Specifically, compared with the DM group, the levels of L-glutamine, L-citrulline, CA, GCA, 3a,7a-dihydroxycholanoic acid, 3a, 6b, 7b-trihydroxy-5b-cholanoic acid, MG [0:0/18:2 (9Z, 12Z)/0:0], PE [22:6 (4Z,7Z,10Z,13Z,16Z, 19Z)/15:0], PG [18:3 (9Z,12Z, 15Z)/22:5 (4Z,7Z,10Z,13Z, 16Z)], TG [18:0/o-18:0/22:5 (7Z,10Z,13Z,16Z, 19Z)], 2-methylbutyroylcarnitine, and 3,5-tetradecadiencarnitine were significantly decreased, while the levels of PE [22:1 (13Z)/22:2 (13Z, 16Z)], PG [16:0/18:3 (9Z,12Z, 15Z)], and TG [20:0/18:3 (9Z,12Z,15Z)/20:2n6] were significantly increased in the MET group. More differential metabolites were found between INS and DM groups, such as L-glutamic acid, L-isoleucine, L-leucine, L-valine, CA, DCA, 3a,7a-ddihydroxycholanoic acid, 3a,6b, 7b-trihydroxy-5b-cholanoic acid, 3-oxocholic acid, lysoPC [16:1 (9Z)], lysoPC(17:0), lysoPC(P-16:0), lysoPE (24:0/0:0), PC [18:1 (11Z)/18:1 (11Z)], PC[18:1 (11Z)/18:3 (9Z,12Z, 15Z)], PC [18:4 (6Z,9Z,12Z, 15Z)/18:4 (6Z,9Z,12Z, 15Z)], PC[20:5 (5Z,8Z,11Z,14Z, 17Z)/14:0], PE [18:4 (6Z,9Z,12Z, 15Z)/20:5 (5Z,8Z,11Z,14Z, 17Z)], PE [P-16:0/14:1 (9Z)], PE (P-16:0e/0:0), PE-NMe2 [16:0/18:1 (9Z)], PG (16:0/16:0), PG [18:3 (6Z,9Z, 12Z)/16:1 (9Z)], PI[16:1 (9Z)/18:1 (11Z)], PI[18:1 (9Z)/18:3 (9Z,12Z, 15Z)], PS(14:0/16:0), TG (16:1 (9Z)/18:0/20:0)(iso6),TG [18:1 (9Z)/24:0/18:3 (6Z,9Z, 12Z)], TG (20:0/14:0/o-18:0), TG (22:0/22:0/o-18:0), 11Z-octadecenylcarnitine, 2-hydroxymyristoylcarnitine, 2-hydroxylauroylcarnitine, 3,5-tetradecadiencarnitine, 3-hydroxy-9-hexadecenoylcarnitine, and trans-2-tetradecenoylcarnitine.

**TABLE 2:** The information of metabolites selected as biomarkers characterized in serum profiles and their taxonomy.

Metabolites	m/z	Rt	DM/CON	MET/DM	INS/DM	Taxonomy
L-Glutamic acid	148.0590	420.2050	↑*	—	↓#	L-alpha-amino acids
L-Glutamine	169.0572	355.0700	↑*	↓##	—	L-alpha-amino acids
L-Isoleucine	130.0860	295.5550	↑*	—	↓##	L-alpha-amino acids
L-Leucine	132.1004	276.8655	↑*	—	↓##	L-alpha-amino acids
L-Valine	159.1111	249.4830	↑*	—	↓##	L-alpha-amino acids
L-Citrulline	176.1022	371.7170	↑*	↓#	—	L-alpha-amino acids
Cholic acid	373.2724	209.8450	↑*	↓#	↓#	Bile acids and derivatives
Deoxycholic acid	391.2828	148.0540	↑*	—	↓#	Bile acids and derivatives
Glycocholic acid	466.3147	234.0415	↑**	↓#	—	Bile acids and derivatives
3a,7a-Dihydroxycholanoic acid	427.2578	159.4030	↑*	↓#	↓#	Bile acids and derivatives
3a,6b,7b-Trihydroxy-5b-cholanoic acid	409.2925	209.7790	↑*	↓#	↓##	Bile acids and derivatives
3-Oxocholic acid	405.2613	136.9160	↑**	—	↓##	Bile acids and derivatives
LysoPC [16:1 (9Z)]	494.3207	111.2680	↑*	—	↓#	Glycerophospholipids
LysoPC (17:0)	544.3123	175.0980	↓**	—	↓##	Glycerophospholipids
LysoPC (P-16:0)	480.3422	167.6570	↓**	—	↓##	Glycerophospholipids
LysoPE (24:0/0:0)	566.4144	168.4340	↓*	—	↓##	Glycerophospholipids
MG [0:0/18:2 (9Z,12Z)/0:0]	337.2718	233.2635	—	↓##	↓##	Monoacylglycerides
PC [18:1 (11Z)/18:1 (11Z)]	844.5764	123.3900	↑*	—	↓##	Glycerophospholipids
PC [18:1 (11Z)/18:3 (9Z,12Z,15Z)]	782.5684	119.1020	↑**	—	↓##	Glycerophospholipids
PC [18:4 (6Z, 9Z, 12Z, 15Z)/18:4 (6Z, 9Z, 12Z, 15Z)]	812.4455	39.7520	↑*	—	↓#	Glycerophospholipids
PC [20:5 (5Z, 8Z, 11Z, 14Z, 17Z)/14:0]	752.5187	124.7615	↑**	—	↓##	Glycerophospholipids
PE [18:4 (6Z, 9Z, 12Z, 15Z)/20:5 (5Z, 8Z, 11Z, 14Z, 17Z)]	794.9319	340.0400	↑**	—	↓#	Glycerophospholipids
PE [22:1 (13Z)/22:2 (13Z, 16Z)]	417.3330	47.3170	↑*	↑#	—	Glycerophospholipids
PE [22:6 (4Z, 7Z, 10Z, 13Z, 16Z, 19Z)/15:0]	750.5032	125.3440	↑*	↓#	—	Glycerophospholipids
PE [P-16:0/14:1 (9Z)]	646.9096	338.7110	↑**	—	↓##	Glycerophospholipids
PE (P-16:0e/0:0)	460.2674	36.5820	↑*	—	↓#	Glycerophospholipids
PE-NMe2 [16:0/18:1 (9Z)]	745.0469	26.0570	↑*	—	↓#	Glycerophospholipids
PG (16:0/16:0)	721.9525	339.4390	↑*	—	↓#	Glycerophospholipids
PG [16:0/18:3 (9Z, 12Z, 15Z)]	745.5051	239.5230	—	↑##	—	Glycerophospholipids
PG [18:3 (6Z, 9Z, 12Z)/16:1 (9Z)]	741.9493	340.0140	↑*	—	↓#	Glycerophospholipids
PG [18:3 (9Z, 12Z, 15Z)/22:5 (4Z, 7Z, 10Z, 13Z, 16Z)]	820.0494	338.6380	—	↓##	↓##	Glycerophospholipids
PI[16:1 (9Z)/18:1 (11Z)]	852.5577	180.2530	↓**	—	↓#	Glycerophospholipids
PI[18:1 (9Z)/18:3 (9Z,12Z,15Z)]	876.5553	176.5865	↓*	—	↓#	Glycerophospholipids
PS(14:0/16:0)	730.8923	339.1120	↑*	—	↓##	Glycerophospholipids
TG [14:1 (9Z)/15:0/20:4 (8Z,11Z,14Z,17Z)]	811.6628	155.8800	↓*	—	↓#	Glycerolipids
TG [16:1 (9Z)/18:0/20:0] (iso6)	940.8009	344.5030	↑*	—	↓#	Glycerolipids
TG [18:0/o-18:0/22:5 (7Z,10Z,13Z,16Z,19Z)]	924.5365	209.4260	—	↓#	↓#	Glycerolipids
TG [18:1 (9Z)/24:0/18:3 (6Z,9Z,12Z)]	984.9234	338.6735	↑**	—	↓##	Glycerolipids
TG (20:0/14:0/o-18:0)	850.4403	338.6760	↑*	—	↓#	Glycerolipids
TG [20:0/18:3 (9Z,12Z,15Z)/20:2n6]	922.4837	114.7160	↓*	↑#	—	Glycerolipids
TG (22:0/22:0/o-18:0)	1,103.8635	338.7090	↑**	—	↓##	Glycerolipids
11Z-Octadecenylcarnitine	426.3567	152.4600	↑*	—	↓#	Acylcarnitine
2-Hydroxylauroylcarnitine	360.2727	191.5340	↑**	—	↓##	Acylcarnitine
2-Methylbutyroylcarnitine	246.1686	223.4530	↓*	↓#	—	Acylcarnitine
3,5-Tetradecadiencarnitine	368.2777	162.5400	↑*	↓##	↓##	Acylcarnitine
3-Hydroxy-9-hexadecenoylcarnitine	414.3194	179.6235	↑**	—	↓##	Acylcarnitine
Trans-2-Tetradecenoylcarnitine	370.2937	159.9770	↑**	—	↓##	Acylcarnitine

### Serum Bile Acids

**FIGURE 5:**
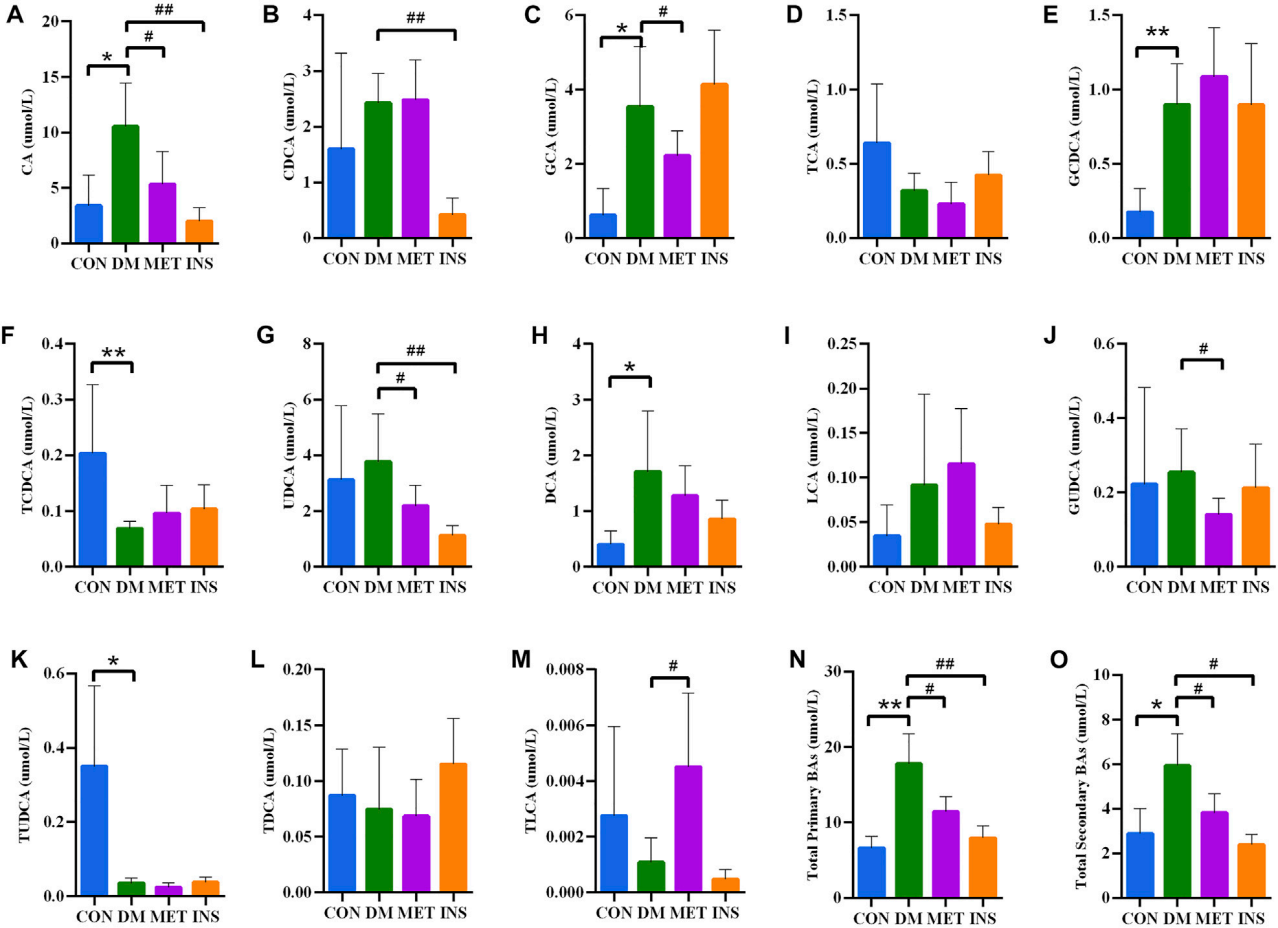
Serum BA concentrations in rats of four groups. **(A)** CA, cholic acid; **(B)** CDCA, chenodeoxycholic acid; **(C)** GCA, glycocholic acid; **(D)** TCA, taurocholic acid; **(E)** GCDCA, glycochenodeoxycholic acid; **(F)** TCDCA, taurochenodeoxycholic acid; **(G)** UDCA, ursodeoxycholic acid; **(H)** DCA, deoxycholic acid; **(I)** LCA, lithocholic acid; **(J)** GUDCA, glycoursodeoxycholic acid; **(K)** TUDCA, Tauroursodeoxycholic acid; **(L)** TDCA, taurodeoxycholic acid; **(M)** TLCA, taurolithocholic acid; **(N)** Total primary BAs; **(O)** Total secondary BAs. *p < 0.05, **p < 0.01 vs. CON group. # *p* < 0.05, ## *p* < 0.01 vs. DM group.

Thirteen BAs were detected in four groups, including both primary BAs (CA, CDCA, GCA, GCDCA, TCA and TCDCA) and secondary BAs (DCA, UDCA, LCA, TLCA, TDCA, GUDCA, and TUDCA) (Figure 5). Compared with the CON group, the serum levels of both primary and secondary BAs were remarkably increased in the DM group. In particular, serum levels of CA, GCA, GCDCA, and DCA were significantly higher, while the levels of TUDCA and TCDCA were lower in the DM group compared with the CON group. No significant differences were detected in serum levels of UDCA, CDCA, TCA, GUDCA, TDCA, LCA, and TLCA between the DM and CON groups. Metformin treatment could significantly decrease the serum levels of CA, GCA, UDCA, and GUDCA, while such treatment increased the level of TLCA in DM rats. Insulin treatment also significantly decreased the level of CA, UDCA, and CDCA.

### Associations Between the Intestinal Microbiota and Serum Metabolites

**FIGURE 6:**
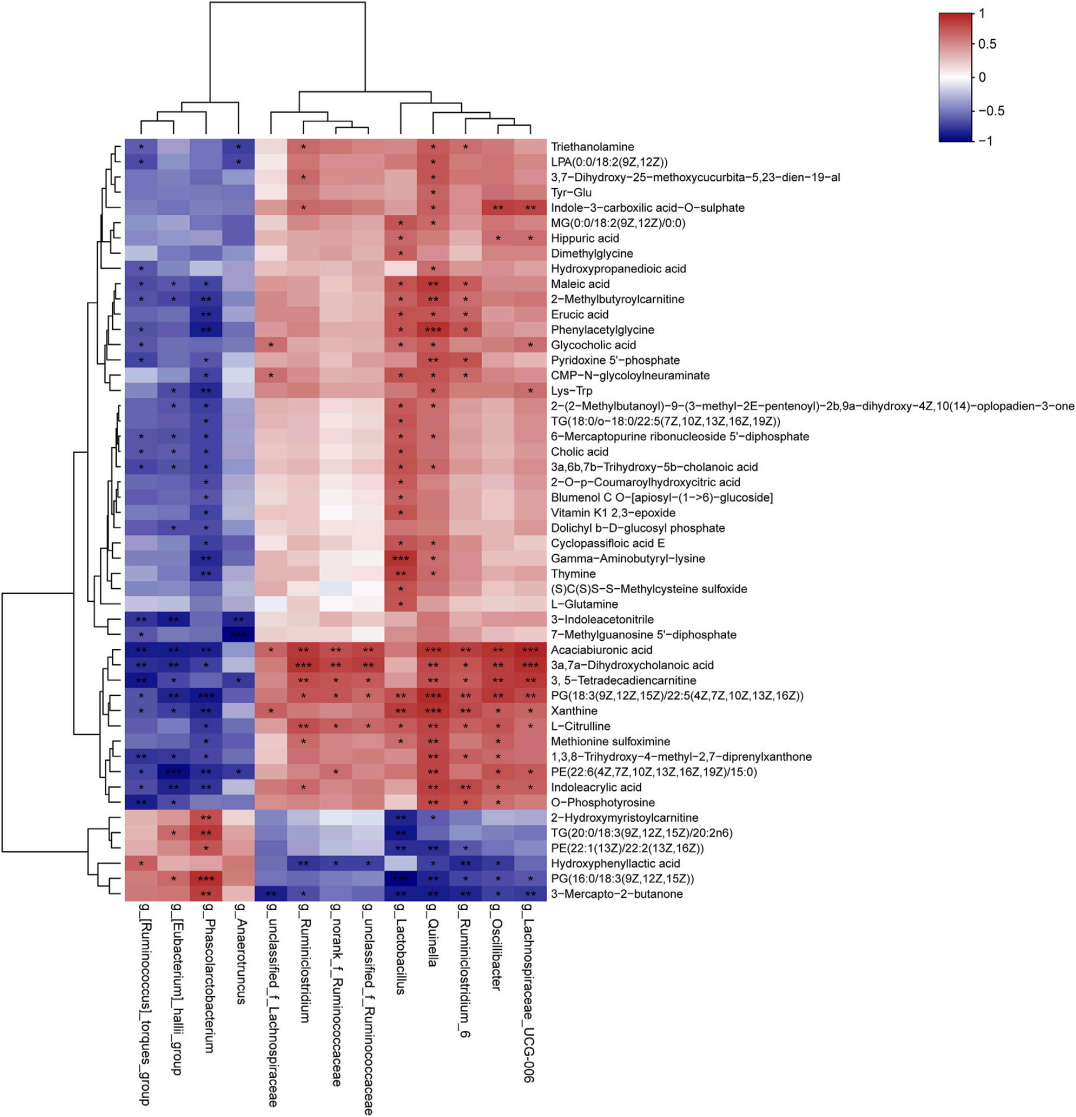
Correlation analysis of the gut microbiome and serum metabolites. The results of Spearman’s correlation between 13 differential genera (MET vs. DM) and 50 differential metabolites (MET vs. DM) were presented as a heatmap. *p < 0.05, **p < 0.01, ***p < 0.001 denoted statistical significance between bacterial taxa and metabolites.

In the present study, we assessed the relationships between the intestinal microbiota and serum differential metabolites identified between DM and MET rats using Spearman’s correlation analysis (Figure 6). L-glutamine and L-citrulline were positively associated with the relative abundance of *Lactobacillus* (*p* < 0.05), and the corresponding r values were 0.68 and 0.66, respectively. Besides, L-citrulline also exhibited a positive correlation with the relative abundances of *Ruminiclostridium*, *norank_f_Ruminococcaceae*, *unclassified_f_Ruminococcaceae*, *Quinella*, *Ruminiclostridium_6*, *Oscillibacter*, and *Lachnospiraceae_UCG-006* (*p* < 0.05, r = 0.60–0.75), and it had a negative correlation with *Phascolarctobacteriu* (*p* < 0.05, *r* = −0.70). CA, GCA, and 3a,6b, 7b-trihydroxy-5b-cholanoic acid showed significant positive correlations with the relative abundance of *Lactobacillus* (*p* < 0.05, *r* = 0.61–0.69). CA, 3a,7a-dihydroxycholanoic acid and 3a, 6b, 7b-trihydroxy-5b-cholanoic acid showed negative correlations with the relative abundances of (*Ruminococcus*)*_torques_group*, (*Eubacterium*)*_hallii_group*, and *Phascolarctobacterium*. LPA (0:0/18:2 (9Z, 12Z)), MG (0:0/18:2 (9Z, 12Z)/0:0), PG [18:3 (9Z,12Z, 15Z)/22:5 (4Z,7Z,10Z,13Z, 16Z)] and PE [22:6 (4Z,7Z,10Z,13Z,16Z, 19Z)/15:0] showed moderate-to-high positive association with the abundance of *Quinella* (*p* < 0.05, r = 0.60–0.87), while PE [22:1 (13Z)/22:2 (13Z, 16Z)] and PG [16:0/18:3 (9Z,12Z, 15Z)] showed high negative correlation with *Quinella* (*p* < 0.01, r = −0.74–0.76). Moreover, 3, 5-tetradecadiencarnitine displayed high positive associations with *Ruminiclostridium_6*, *Quinella*, *Oscillibacter* and *Lachnospiraceae_UCG-006* (*p* < 0.01, r = 0.71–0.80), while they had negative association with (*Ruminococcus*)*_torques_group* (*p* < 0.01, r = −0.82).

**FIGURE 7:**
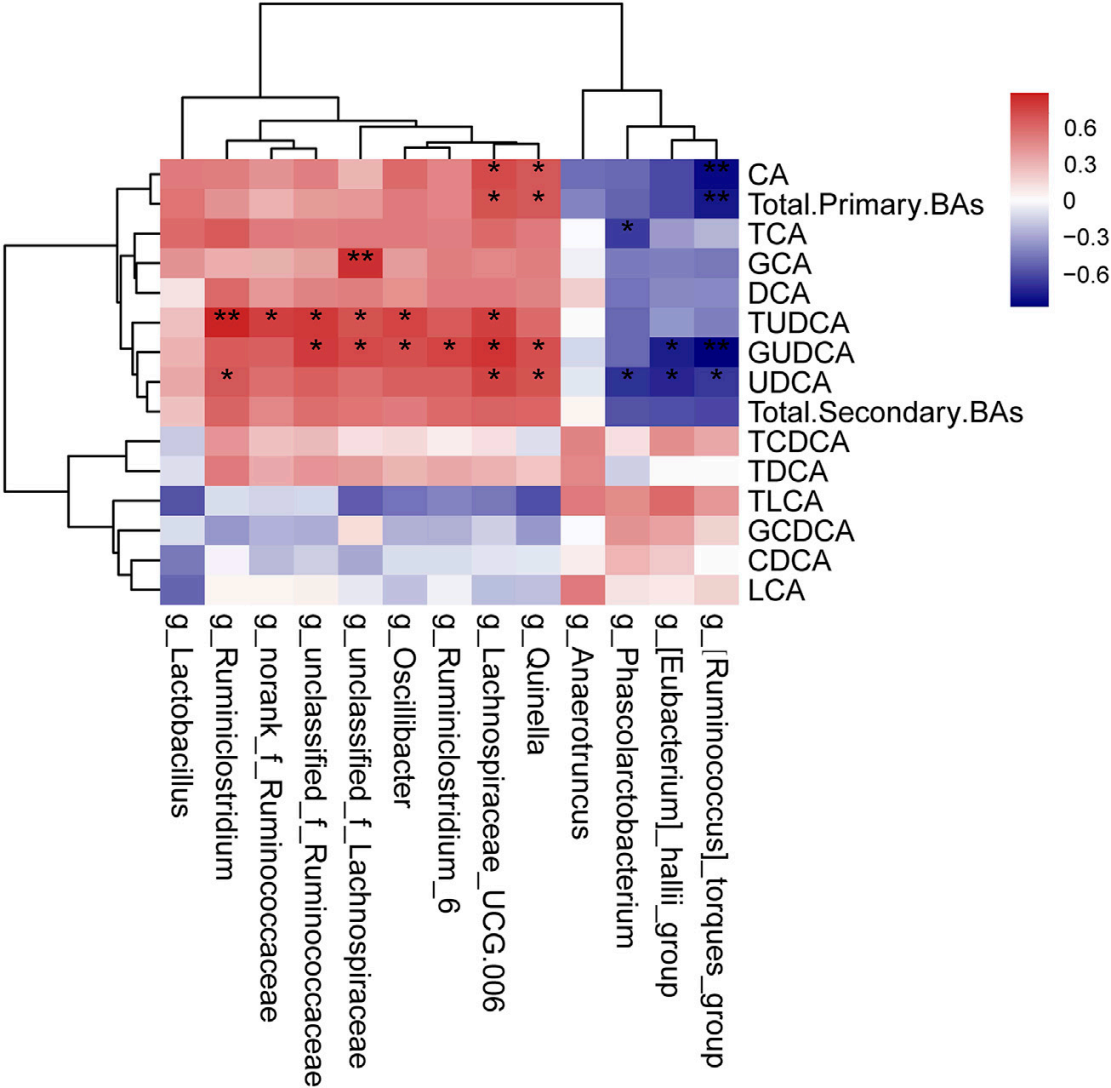
Correlation analysis of the gut microbiome and serum BAs. The results of Spearman’s correlation between 13 differential genera (MET vs. DM) and BAs (MET vs. DM) were presented as a heatmap. *p < 0.05, **p < 0.01 denoted statistical significance between bacterial taxa and BAs.

The relationships between the intestinal microbiota and serum BAs in DM and MET rats were also assessed using Spearman’s correlation analysis (Figure 7). CA and total primary BAs were positively associated with the relative abundances of *Quinella* and *Lachnospiraceae_UCG-006* (*p* < 0.05), while they were negatively associated with (*Ruminococcus*)*_torques_group* (*p* < 0.01, r = 0.71–0.75). TCA exhibited a negative correlation with the relative abundances of *Phascolarctobacteriu.* GCA showed sigenificant positive correlation with the relative abundance of *unclassified_f_Lachnospiraceae* (*p* < 0.01, r = 0.72). TUDCA, GUDCA, and UDCA displayed positivecorrelation with the relative abundances of several genera, such as *Ruminiclostridium*, *norank_f_Ruminococcaceae*, *unclassified_f_Ruminococcaceae*, *unclassified_f_Lachnospiraceae*, *Oscillibacter*, *Ruminiclostridium_6*, *Lachnospiraceae_UCG-006*, and *Quinella.* Besides, GUDCA and UDCA had negative correlations with the relative abundances of (*Ruminococcus*)*_torques_group* and (*Eubacterium*)*_hallii_group*.

## Discussion

In the present study, we showed that both metformin and insulin treatment reduced the blood glucose level, ameliorated the lipid metabolism, changed the composition of gut microbiota, and altered the serum metabolome in T2DM rats induced by the combination of STZ and HFD. Metformin treatment for 3 weeks partially decreased the levels of blood glucose, TC, TG, and LDL-C in DM rats, and the effectiveness was weaker compared with the insulin treatment. Metformin and insulin treatment altered the gut microbiota and metabolome profiles differently, indicating that different mechanisms were involved in the two types of pharmacotherapy.

Firmicutes and Bacteroidetes are the two dominant phyla in the gut microbiota, and the Bacteroidetes/Firmicutes ratio has been previously suggested as a marker of metabolic disease. Accumulating evidence has confirmed that diabetes and obesity can decrease the ratio of Bacteroidetes/Firmicutes in humans and animals (Everard and Cani, 2013; Gurung et al., 2020). Several investigations have shown that metformin treatment can elevate the ratio of Bacteroidetes/Firmicutes (Ryan et al., 2020; Zhang and Hu, 2020). In the present study, we also found that metformin and insulin both increased the ratio of Bacteroidetes/Firmicutes. It was noticeable that the relative abundance of phylum Actinobacteria was remarkably different among the four groups. Diabetes elevated the relative abundance of phylum Actinobacteria, which was regulated oppositely by the treatment of metformin or insulin. Metformin further increased the abundance, while insulin decreased the relative abundance of Actinobacteria. A study regarding the intestinal microbiome of Chinese T2DM patients has shown that the relative abundance of Actinobacteria in T2DM patients treated with metformin is markedly greater compared with the untreated T2DM patients, which is in agreement with our data (Zhang F. et al., 2019).

At the genus level, 13 genera changed significantly after metformin treatment, while only three changed after insulin treatment, indicating the greater influence of metformin on the gut microbiota. Among these genera, the abundances of short-chain fatty acid (SCFA)-producing bacteria, such as *Phascolarctobacterium*, *Anaerotruncus*, (*Eubacterium*)*_hallii_group*, and (*Ruminococcus*)*_torques_group* were significantly increased after metformin treatment. SCFAs can activate intestinal gluconeogenesis and have beneficial effects on glucose and energy homeostasis (Larsen et al., 2010). SCFAs can be produced by certain bacteria. For example, propionic acid can be produced by *Phascolarctobacterium* (Reichardt et al., 2014), and butyric acid can be produced by *Eubacterium*, *Roseburia*, and *Faecalibacterium* (Louis et al., 2004). A lot of studies have reported that metformin regimen can elevate the abundances of SCFA-producing bacteria in diabetic animals and patients (Lee and Ko, 2014; Shin et al., 2014; Forslund et al., 2015; De La Cuesta-Zuluaga et al., 2017; Wu et al., 2017; Lee et al., 2018). The abundance of *Ruminococcus* was also found increased in db/db mice and C57BL/6J mice by metformin treatment (Bornstein et al., 2017; Zhang W. et al., 2019; Ahmadi et al., 2020). Zhang et al. have reported that metformin treatment can increase the abundance of *Phascolarctobacterium* in Wistar rats fed with HFD (Zhang et al., 2015). We also found that the abundances of two genera *unclassified_f_Lachnospiraceae* and *Lachnospiraceae_UCG-006*, which belong to the family *Lachnospiraceae,* were decreased in the MET group compared with the CON group. It has been reported that the abundance of *Lachnospiraceae* is increased in obese mice fed by HFD (Li et al., 2021), while it is decreased in women with a vegetarian diet (Barrett et al., 2018). Liraglutide, a glucagon-like peptide-1 (GLP-1) analog, significantly increases the abundances of *Lachnospiraceae_UCG-001* and *Lachnospiraceae_NK4A136_group nonalcoholic* in db/db mice with nonalcoholic fatty liver (Liu et al., 2020), suggesting that the decrease of *Lachnospiraceae* is beneficial for T2DM. The abundance of *unclassified Lachnospiraceae* is markedly decreased in metformin-treated obese patients compared with the metformin-naive obese patients (Hiel et al., 2020). Similar results are also observed in Wistar rats fed with HFD and T2DM Sprague-Dawley rats, showing that the abundances of *Lachnospiraceae_incertae_sedis* and *Lachnospiraceae NK4A136* are decreased after metformin administration (Zhang et al., 2015; Cui et al., 2019). Ryan et al. have reported that the abundance of *Ruminococcus* is decreased by metformin treatment in C57BL/6 mice fed with HFD (Ryan et al., 2020). We also found that the abundances of *norank_f_Ruminococcaceae* and *unclassified_f_Ruminococcaceae* were reduced by metformin. Elbere et al. have shown that the metformin treatment can elevate the abundance of *Oscillibacter* in both healthy nondiabetic individuals and T2DM patients (Elbere et al., 2020), which is opposite to our results. The alteration of *Lactobacillus* in our present work was also inconsistent with previous findings, in which its abundance is increased by metformin treatment in obese or diabetic rodents (Zhang et al., 2015; Zhang M. et al., 2019; Cui et al., 2019).

Amino acids promote the production of endogenous glucose as substrates of gluconeogenesis (Schutz, 2011). Insulin resistance is associated with higher levels of branched-chain amino acids (BCAAs), aromatic amino acids, and glutamate/glutamine (Tai et al., 2010). Among the serum differential metabolites between CON and DM groups, the levels of L-glutamic acid, L-glutamine, L-citrulline, and BCAAs (L-isoleucine, L-leucine, and L-valine) were all remarkably increased in the DM group compared with the CON group. After metformin treatment, the serum levels of L-glutamine and L-citrulline were decreased. It has been reported that metformin regulates ammonia homeostasis by controlling glutamine metabolism in the enterocyte, exerting an indirect regulatory effect on both the uptake and degradation of glutamine (Gil-GÓmez et al., 2018). Adam et al. have assessed 353 metabolites in fasting serum samples from T2DM patients who are treated with metformin or without anti-diabetic medication and found that citrulline is significantly lower in metformin-treated T2DM patients compared with those not receiving anti-diabetic medication. Citrulline is also confirmed to be significantly reduced in patients receiving metformin treatment for 7 years. Furthermore, lower citrulline levels in plasma, skeletal muscle, and adipose tissue are validated in mice receiving metformin (Adam et al., 2016). Moreover, the plasma concentrations of citrulline and arginine in overweight/obese adults with impaired fasting glucose can be decreased after 3 months of metformin plus pioglitazone regimen (Irving et al., 2015). Besides, acute administration of metformin decreases the concentration of plasma citrulline in non-diabetic African Americans (Rotroff et al., 2016). Citrulline plays a prominent role in nitric oxide biosynthesis and the urea cycle. The potential mechanism underlying metformin’s effect on citrulline metabolism is related to the role of metformin in cellular and systemic nitric oxide and/or urea biosynthesis in individuals with T2DM (Irving and Spielmann, 2016). Many studies have confirmed that obesity and insulin resistance are associated with elevated circulating levels of BCAAs. BCAA and related metabolites are widely accepted as the most efficient biomarkers of obesity, insulin resistance, and T2DM in human (Knebel et al., 2016; Bloomgarden, 2018; White et al., 2021). In the present study, diabetes dramatically elevated the levels of BCAAs, which was consistent with previous studies. The change of BCAA level was not significant between the DM and MET groups, indicating that the metformin treatment might not affect on the BCAA metabolism. However, after insulin treatment, the levels of all three BCAAs, including L-isoleucine, L-leucine, and L-valine, were significantly decreased. The difference in effect on BCAA metabolism might be one of the distinctions in the action mechanism between metformin and insulin.

Acylcarnitines (ACs) function as carnitine esters of fatty acids that have entered the mitochondria. Lately, ACs are suggested as biomarkers of insulin resistance and metabolic inflexibility in humans (Mihalik et al., 2010; Ramos-Roman et al., 2012). Previous studies have indicated that the fatty acid oxidation rate exceeds the tricarboxylic acid cycle, thus resulting in the deposition of intermediary metabolites such as ACs (Muoio and Neufer, 2012; Schooneman et al., 2013). Makarova et al. have reported that insulin secretion upon glucose treatment reduces the plasma levels of long-chain acylcarnitin of normal mice by 30% (Makarova et al., 2019). We found that several ACs exhibited significant differences between the DM and CON groups. The serum levels of 2-methylbutyroylcarnitine, 11Z-octadecenylcarnitine, 2-hydroxylauroylcarnitine, 3,5-tetradecadiencarnitine, 3-hydroxy-9-hexadecenoylcarnitine and trans-2-tetradecenoylcarnitine were higher in diabetic rats compared with the control rats, and insulin treatment could decrease the levels of all these ACs. The effect of metformin on ACs seemed weaker compared with insulin, and metformin treatment only decreased the levels of 2-methylbutyroylcarnitine and 3,5-tetradecadiencarnitine. Paul et al. have also reported that metformin reduces the levels of several ACs in metabolically dysfunctional mice (Ryan et al., 2020).

Lipid metabolism plays a fundamental role in the pathogenesis of diabetes. Dyslipidemia can promote the insulin resistance process, and further aggravate T2DM. Many studies have shown that elevated lipotoxicity, such as enhanced synthesis of fatty acids, sphingolipids and phospholipids, is associated with the pathogenesis of diabetes (Zhu et al., 2011; Floegel et al., 2013; Meikle et al., 2013; Shui et al., 2013; Zhao et al., 2013; Knebel et al., 2016; Tonks et al., 2016). It has been reported that there is a positive correlation between T2DM ceramide, and its precursor dihydroceramide, as well as phosphatidylethanolamine, phosphatidylglycerol and phosphatidylinositol (Meikle et al., 2013). TG is one of the high-risk factors for T2DM, the level of which should be strictly controlled in T2DM patients. We found that a lot of lipids, including PC, PE, PG, PI, PS and TG, were all significantly higher in T2DM rats compared with the normal rats, and insulin treatment could alleviate most of them. Metformin has beneficial effects on improving lipid metabolism, resulting in a reduction of chylomicrons by up to 50% in T2DM patients (He, 2020). Controversial conclusions have been obtained on the effects of metformin on lipid metabolism. For instance, Safai et al. have shown that T2DM patients treated with metformin have higher levels of five lysophosphatidylethanolamines (LysoPEs) compared with metformin-naïve patients (Safai et al., 2018), while Wanninger et al. have found that the levels of PC, lysoPC, phosphatidylserine, and sphingomyelin (derived from PC) were lower in metformin-exposed hepatocytes (Wanninger et al., 2008). It has been believed that metformin reduces the content of hepatic lipid by activating AMPK, thereby ameliorating the situation in hyperglycemia and insulin resistance (Viollet et al., 2012). However, in our present study, metformin treatment showed a weaker influence on this dyslipidemia, and only very few types of lipid were reversed. It was possibly attributed to the short course of metformin treatment, and the effect of metformin on lipid metabolism disorders has not been shown.

As the main element of bile, BAs not only facilitate the digestion and absorption of fat but also are involved in glycolipid and energy metabolism. BAs are cholesterol catabolites that are mainly synthesized in the liver, in which CA and CDCA are the two primary BAs generated. Following hepatic synthesis, BAs are secreted into bile as glycine or taurine conjugates. BAs are actively reabsorbed by enterocytes in the terminal ileum to hepatocytes, where they are taken up and reused. A small proportion of BAs is modified by intestinal microbiota and passively reabsorbed in the colon. Primary BAs can be metabolized to secondary BAs by gut bacteria. In the intestine, a part of conjugated CA and CDCA is de-conjugated by gut bacterial bile salt hydroxylase (BSH) to form DCA and LCA. In addition, small amounts of CDCA are converted to UDCA by gut bacterial 7β-hydroxysteroid dehydrogenase (Ferrell and Chiang, 2019). It has been demonstrated that BAs can take part in both glucose metabolism and energy regulation, mostly via the activation of the farnesoid X receptor (FXR) and the G protein-coupled BA receptor 1 (BA membrane-type receptor TGR5). A lot of studies have shown that hepatic insulin resistance and hyperglycemia increase BA synthesis, resulting in alterations in BA composition. For example, it has been reported that diabetic (db/db) mice have a larger total BA pool size than wild-type control animals (Chen et al., 2016). The levels of postprandial TBA, CA, CDCA, DCA and UDCA were greater in T2DM patients compared with healthy controls (Sonne et al., 2016). In our current work, both metformin and insulin could partially recover the increased TBA in diabetic rats. Moreover, several BAs changed significantly among different groups. For example, the levels of CA, DCA, GCA, CDCA, 3a,7a-dihydroxycholanoic acid, 3a,6b,7b-trihydroxy-5b-cholanoic acid and 3-oxocholic acid were higher in the DM group compared with the CON group. The level of CA, GCA, 3a,7a-dihydroxycholanoic acid and 3a,6b,7b-trihydroxy-5b-cholanoic acid were lower in the MET group compared with the DM group. Besides, we further determine the levels of 13 types of BAs, including six primary BAs and seven secondary BAs, using LC-MS/MS. Compared with the DM group, metformin and insulin treatment could both decrease the levels of total primary BAs and total secondary BAs. In addition, metformin could decrease the levels of CA, GCA, UDCA, and GUDCA, while it increased the level of TLCA. The levels of CA, CDCA and UDCA in DM rats were decreased after insulin administration. Metformin can ameliorate glucose metabolism by modulating the TBA level in the serum of diabetic animals. It has been reported that metformin treatment increases the level of BSH produced by the gut microbiota in diabetic mice (Wu et al., 2017). Sun et al. have shown that metformin changed the level of GUDCA by modulating the gut microbiota (such as inhibition of *Bacteroides fragilis* growth), thereby suppressing the FXR signaling pathway to decrease blood glucose and maintain blood glucose homeostasis. It has been hypothesized that metformin reduces the reabsorption of BA in the distal ileum, resulting in increased bile salt concentration within the colon, which may explain the impacts of metformin on the colonic microbiota (Carter et al., 2003).

Collectively, we, for the first time, evaluated the impacts of metformin on the gut microbiota and assessed the interplay between gut microbiota and host metabolism in T2DM rats induced by a combination of STZ and HFD. The above-mentioned effects of metformin were also compared with insulin treatment to further investigate the different therapeutic mechanisms between metformin and insulin. Compared with insulin treatment, metformin showed greater influences on the composition of the gut microbiota, while it had a weaker impact on serum metabolites. The therapeutic mechanisms of metformin on diabetic rats were likely associated with restoration of the dysbiosis of gut microbiota and regulation of the disorder of amino acids (L-glutamine and L-citrulline), glycerophospholipids/glycerolipids, acylcarnitine (3,5-tetradecadiencarnitine), and BAs. Taken together, regulating the BA levels might be a critical mechanism underlying the therapeutic effects of metformin on diabetes. Our findings provided valuable insights in to the latent mechanism of metformin.

## Data Availability

The raw sequencing data in our study have been deposited in the BioProject: PRJNA774924, https://www.ncbi.nlm.nih.gov/bioproject/?term=PRJNA774924.
