# Supplementary material for: Comparative Evaluation of the Effect of Metformin and Insulin on Gut Microbiota and Metabolome Profiles of Type 2 Diabetic Rats Induced by the Combination of Streptozotocin and High-Fat Diet
Source: Front Pharmacol. 2022 Jan 3;12:794103. doi: 10.3389/fphar.2021.794103 (PMC8762251; doi:10.3389/fphar.2021.794103)
Supplement: Supplementary file 2 [file Table1.DOCX]

**Supplementary Table 1** The precursor ion and product ion mass, declustering potentials (DP), collision energies (CE), retention time (RT) and liner range of each bile acid

| Bile acid | Precursor ion (Da) | Product ion (Da) | DP (V) | CE (V) | RT (min) | Liner range (μg/mL) |
| --- | --- | --- | --- | --- | --- | --- |
| CA | 407.1 | 407.1 | -20 | -165 | 1.25 | 4-1000 |
|  | 407.1 | 343.3 | -48 | -165 | 1.25 |  |
| GCA | 464.3 | 74.1 | -78 | -150 | 1.51 | 20-5000 |
|  | 464.3 | 464.3 | -15 | -150 | 1.51 |  |
| GUDCA | 448.3 | 74.0 | -70 | -150 | 1.30 | 20-5000 |
|  | 448.3 | 386.1 | -50 | -150 | 1.30 |  |
| TCA | 514.2 | 80.0 | -115 | -210 | 1.76 | 10-2000 |
|  | 514.3 | 124.1 | -67 | -200 | 1.76 |  |
| TUDCA | 498.3 | 498.3 | -35 | -210 | 1.60 | 1-250 |
|  | 498.3 | 80.0 | -115 | -210 | 1.60 |  |
| UDCA | 391.3 | 391.3 | -25 | -165 | 1.10 | 4-1000 |
|  | 391.3 | 355.2 | -48 | -165 | 1.10 |  |
| CDCA | 391.3 | 391.3 | -24 | -160 | 2.30 | 4-1000 |
|  | 391.3 | 373.2 | -46 | -160 | 2.30 |  |
| GCDCA | 448.3 | 74.0 | -72 | -150 | 2.75 | 20-5000 |
|  | 448.3 | 386.4 | -47 | -150 | 2.75 |  |
| TCDCA | 498.3 | 498.3 | -35 | -200 | 3.10 | 4-1000 |
|  | 498.3 | 80.0 | -115 | -200 | 3.10 |  |
| TDCA | 498.3 | 79.8 | -115 | -210 | 3.42 | 4-1000 |
|  | 498.3 | 355.1 | -67 | -210 | 3.42 |  |
| DCA | 391.3 | 391.3 | -25 | -165 | 2.68 | 4-1000 |
|  | 391.3 | 345.2 | -46 | -165 | 2.68 |  |
| LCA | 375.3 | 375.3 | -35 | -190 | 4.32 | 1-250 |
|  | 375.3 | 357.3 | -48 | -210 | 4.32 |  |
| TLCA | 482.2 | 79.9 | -115 | -210 | 5.24 | 1-250 |
|  | 482.3 | 124.0 | -63 | -180 | 5.24 |  |
| GCA-d5 | 469.3 | 469.3 | -15 | -150 | 1.51 |  |
|  | 469.3 | 74.1 | -78 | -150 | 1.51 |  |
| CDCA-d4 | 395.3 | 395.3 | -24 | -180 | 2.30 |  |
| GCDCA-d7 | 455.3 | 75.0 | -70 | -150 | 2.75 |  |
| DCA-d5 | 396.3 | 396.3 | -25 | -165 | 2.68 |  |
| LCA-d4 | 379.3 | 379.3 | -30 | -180 | 4.32 |  |
